# Hypertensive Target Organ Damage in Ghanaian Civil Servants with Hypertension

**DOI:** 10.1371/journal.pone.0006672

**Published:** 2009-08-18

**Authors:** Juliet Addo, Liam Smeeth, David A. Leon

**Affiliations:** Department of Epidemiology and Population Health, London School of Hygiene and Tropical Medicine, London, United Kingdom; Swiss Paraplegic Research, Switzerland

## Abstract

**Background:**

Low levels of detection, treatment and control of hypertension have repeatedly been reported from sub Saharan Africa, potentially increasing the likelihood of target organ damage.

**Methods:**

A cross-sectional study was conducted on 1015 urban civil servants aged≥25 years from seven central government ministries in Accra, Ghana. Participants diagnosed to have hypertension were examined for target organ involvement. Hypertensive target organ damage was defined as the detection of any of the following: left ventricular hypertrophy diagnosed by electrocardiogram, reduction in glomerular filtration rate, the presence of hypertensive retinopathy or a history of a stroke.

**Results:**

Of the 219 hypertensive participants examined, 104 (47.5%) had evidence of target organ damage. The presence of target organ damage was associated with higher systolic and diastolic blood pressure levels. The odds of developing hypertensive target organ damage was five to six times higher in participants with blood pressure (BP)≥180/110 mmHg compared to those with BP<140/90 mmHg, and there was a trend to higher odds of target organ damage with increasing BP (p = 0.001). Women had about lower odds of developing target organ damage compared to men.

**Conclusions:**

The high prevalence of target organ damage in this working population associated with increasing blood pressure, emphasises the need for hypertension control programs aimed at improving the detection of hypertension, and importantly addressing the issues inhibiting the effective treatment and control of people with hypertension in the population.

## Introduction

Hypertension is considered to be a major public health problem on the African continent. It is of immense economic importance because of the high prevalence in urban areas, frequent under-diagnosis and the severity of complications.[1], [2], [3], [4] Of particular concern are the low levels of detection, treatment and control of hypertension reported from studies conducted in sub Saharan Africa (SSA).[5], [6], [7], [8], [9] The reported poor control increases the possibility of developing complications of hypertension with potential damage to the prime target organs of hypertensive damage including the heart, kidney, brain and arterial blood vessels, with a worsening of the prognosis.[10] Most of the disease burden caused by high blood pressure worldwide is reported to be borne by low and middle-income countries including some countries in SSA.[11], [12] In addition, people of black African origin have been identified as having a higher risk of target organ damage compared to white people for a given blood pressure.[13], [14], [15]

Almost all published studies on hypertensive target organ damage from sub Saharan Africa have been hospital-based, potentially capturing hypertensive patients with more severe or symptomatic disease who are more likely to attend hospital clinics regularly. The aim of our study was to determine the prevalence of target organ involvement in Ghanaian civil servants, who had essential hypertension and to examine the factors associated with this.

## Materials and Methods

**Figure 1:**
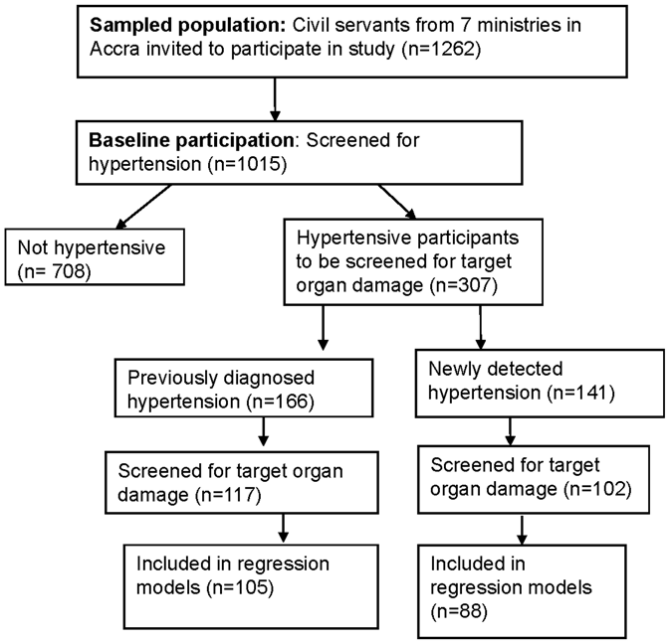
Flow diagram of study activities.

A cross sectional study of hypertension was conducted among civil servants in Accra, the capital city of Ghana between January and September 2006. The study involved seven ministries selected randomly from a list of all 26 civil service ministries in Accra, Ghana. Workers of all employment grades in the central administration offices of these seven ministries aged 25 years and above were invited to participate. A recruitment system was set up whereby an administrative officer from each ministry or department visited, was designated a duty to invite all workers to participate in the study. The workers had been informed ahead of time and were called from their offices to a location, usually the conference room of the ministry on the days of the study. In all 1015 out of 1262 eligible workers from these ministries had their blood pressure (BP) measured using an electronic device (Omron M5I) after participants had been sitting quietly for at least ten minutes. Three measurements were taken at one minute interval on the right arm in a seated position, with arm supported at heart level and feet flat on the floor using an appropriate sized cuff. Hypertension was diagnosed when the mean of the second and third blood pressure (BP) measurements at each of two visits (three weeks apart) was ≥140/90 mmHg or when participants reported use of antihypertensive medication. All civil servants who were diagnosed to have hypertension from this initial screening (307) were invited for further investigations to examine for target organ involvement. These investigations were carried out in the Civil Service Clinic which was centrally located. A flow diagram of participation in study activities is shown in Figure 1. The protocol was approved by the ethics committees of the University of Ghana Medical School and the London School of Hygiene and Tropical Medicine and written informed consent was obtained from each participant before inclusion into the study. Other aspects of the study have been described elsewhere.[16], [17]

Data on age, level of education, duration of hypertension and treatment, history of cigarette smoking, current and past symptoms of cerebrovascular disease, peripheral vascular disease and coronary heart disease were collected by interview using a structured questionnaire. Participants were classified, based on level of blood pressure, into Grade 1 hypertension (mild) if systolic blood pressure was between 140 and 159 mmHg or diastolic blood pressure was between 90 and 99 mmHg; Grade 2 hypertension (moderate) if systolic blood pressure was between 160 and 179 mmHg or diastolic between 100 and 109 mmHg; Grade 3 hypertension (severe) if systolic blood pressure was ≥180 mmHg and diastolic blood pressure was ≥110 mmHg.[18], [19] In terms of education, participants were classified as “Primary” where they had completed 9 years of education or less, “Secondary” where they had completed Senior Secondary School comprising 12 years of education; and “Tertiary” where participants had completed university education or higher. Weight was measured using a professional Seca floor scale to the nearest 0.1 kg. Standing height was measured with a Seca stadiometer to the nearest millimeter with the participant standing erect with back straight, heels together, and toes slightly apart at a 60 degree angle. Body mass index was calculated as the weight in kilograms divided by the square of the height in metres.

A venous blood sample was drawn from an antecubital vein after an overnight fast (10–14 hours) into plain vacutainer tubes and centrifuged within one hour, at room temperature (15–24°C) for at least 10 minutes. Plasma glucose, urea and creatinine were measured by enzymatic methods using the Humalyzer Junior Semi-automated Chemistry Analyzer. For proper calibration of the measurement instrument, precision and accuracy of measurements, controls were assayed with each batch of samples using the ‘Humatrol N’ and ‘Humatrol P’ from the ‘Human’ range of reagents. A mid-stream urine sample was collected for biochemistry and microscopy.

Retinal examination was carried out by an experienced ophthalmologist with no prior knowledge of the participants' blood pressure level. Retinal changes associated with hypertension were classified into four grades using the Keith, Wagener, and Barker classification based on the level of severity of the retinal findings.[20] Grade 1 consisted of ‘mild’ generalized retinal arteriolar narrowing; Grade 2 consisted of ‘more severe’ generalized narrowing, focal areas of arteriolar narrowing and arteriovenous nicking; Grade 3 consisted of grades 1 and 2 signs plus the presence of retinal haemorrhages, microaneurysms, hard exudates and cotton-wool spots; Grade 4 also referred to as accelerated (malignant) hypertensive retinopathy, consisted of the signs in the preceding three grades plus optic disk swelling and macular oedema.[20] Stroke was identified through the history of a previous event (including transient ischaemic stroke).

A 12- lead electrocardiogram (ECG) was performed on participants lying supine and flat on a couch in an air- conditioned room at a room temperature of 25°C. The tracings were coded in duplicate manually by two cardiologists with adequate training in coding of ECG and a consensus reached between the two where there was any disagreement. All ECGs were coded on the basis of the Minnesota coding criteria (III_1_ and IV_1–3_ or V_1–3_).[21] Left ventricular hypertrophy was diagnosed on the basis of fulfilment of at least one of the following voltage criteria on ECG: R wave>1.1 mV in aVL; R wave>2.5 mV in V_5_ or V_6_; S wave>2.5 mV in V_1_ or V_2_; sum of S in V_1_ or V_2_ plus R in V_5_ or V_6_>3.5 mV; or sum of R in I and S in III>2.5 mV.[22] There was further classification of ECG strain pattern of ST depression and T-wave inversion where there was evidence of left ventricular hypertrophy.[23], [24]

Chronic kidney disease (CKD) was defined as persistent proteinuria on urinalysis in the absence of urinary tract infection and/or Impaired glomerular filtration rate (<60 ml.min^−1^per 1.73 m^2^).[25], [26] Glomerular filtration rate (GFR) was estimated from serum creatinine levels using the Modification of Diet in Renal Disease (MDRD) formula that also takes into account age, sex and race.[27], [28] Diabetes was defined as a venous fasting plasma glucose of ≥7.0 mmol/l and impaired fasting glucose (IFG) as fasting glucose levels> = 6.1 mmol/l and <7.0 mmol/l. Blood glucose was considered to be normal if fasting plasma glucose (FPG) was <6.1 mmol/L.[29]

All data forms were entered in Excel and were checked for range and internal consistency. Analysis was conducted using STATA Release 9 (Stata Corp, College Station, TX). Statistical tests included the chi squared test for comparison of proportions and student's t-test for normally distributed data. Univariate and logistic regression models were used to examine the associations between any target organ damage and the variables of interest; with age, sex, detection and treatment of hypertension, blood pressure level, level of education, history of smoking and diagnosis of diabetes included in the models that were considered. For the logistic regression analysis hypertensive target organ damage was defined as the detection of one or more of the following: left ventricular hypertrophy diagnosed by ECG, impaired glomerular filtration rate (<60 ml.min^−1^per 1.73 m^2^), the presence of hypertensive retinopathy Grades III and IV or a history of a stroke. We considered the possibility of interaction between some explanatory variables and sex, because of the marked sex differences in their distribution and introduced interaction parameters into the regression models to test for interaction formally using the likelihood ratio test. Tests for trend were determined in the models where necessary, based on regression analysis with the relevant factor entered as a continuous variable. Analyses of the relationship between hypertension and the variables of interest were performed on participants with no missing data in any of the variables included in the models (n = 193).

## Results

A total of 219 out of the 307 civil servants with hypertension invited to participate reported for further investigations (71.3%). All participants were Ghanaians. The participants were significantly older 50.4 years (SD 6.6 years) than the non-participants; 48.1 years (SD 7.7 years) (p-value for difference 0.01). There was no significant difference between the mean systolic (p-value 0.26) and diastolic blood pressures (p-value 0.84) of participants (156.5/95 mmHg) and non-participants (153.4/95.3 mmHg). The general characteristics of the participants and non-participants are shown in table 1. The detection and treatment rates of hypertension were similar in participants and non-participants in both sexes, suggesting that the blood pressure related characteristics of non-participants were not substantially different from that of participants. The mean duration of hypertension among the 116 participants (53%) who provided this information was 5.7 years (95% CI: 4.8–6.7). The prevalence of diabetes among participants was 9.1% (10.0% in males and 7.6% in females) with 17.4% having impaired fasting glucose. The prevalence of smoking among male participants was 6.5% with no reports of any female participant smoking at the time of the study.

**Table 1:** Baseline characteristics of study population.

	Participants			Non Participants		
	Men (140)	Women (79)	All (219)	Men (55)	Women (33)	All (88)
Mean age in years (SD)	50.4 (6.8)	50.4 (6.3)	50.4 (6.6)	48.0 (8.5)	48.3 (6.2)	48.1 (7.7)
Mean SBP in mmHg (SD)	159.2 (19.6)	151.7 (23.9)	156.5 (21.5)	158.6 (22.1)	144.8 (20.6)	153.4 (22.4)
Mean DBP in mmHg (SD)	96.3 (11.4)	92.7 (15.3)	95.0 (13.0)	97.2 (13.8)	92.2 (11.5)	95.3 (13.1)
Mean BMI in kg/m^2^ (SD)	26.1 (4.5)	29.9 (6.0)	27.5 (5.4)	25.1 (4.6)	32.4 (5.6)	27.9 (6.1)
**Level of education%(n)**						
Primary	30.2 (42)	29.1 (23)	29.8 (65)	40.0 (22)	19.4 (28)	32.6 (28)
Secondary	25.9 (36)	41.8 (33)	31.7 (69)	25.5 (14)	58.1 (18)	37.2 (32)
Tertiary	43.9 (61)	29.1 (23)	38.5 (84)	34.6 (19)	22.6 (7)	30.2 (26)
**Smoking% (n)**						
Never	87.7 (121)	98.7 (78)	91.7 (199)	78.2 (43)	100 (33)	86.4 (76)
Current	6.5 (9)	0	4.2 (9)	12.7 (7)	0	8.0 (7)
Ex-smoker	5.8 (8)	1.3 (1)	4.2 (9)	9.1 (5)	0	5.7 (5)
**Detection of hypertension%(n)**						
Previously detected	42.9 (60)	72.2 (57)	53.4 (117)	43.6 (24)	75.8 (25)	55.7 (49)
Newly detected	57.1 (80)	27.9 (22)	46.6 (102)	56.4 (31)	24.2 (8)	44.3 (39)
**Treatment of hypertension%(n)**						
No treatment	76.4 (107)	54.4 (43)	68.5 (150)	80.0 (44)	51.5 (17)	69.3 (61)
Current treatment	23.6 (33)	45.6 (36)	31.5 (69)	20.0 (11)	48.5 (16)	30.7 (27)
**Duration of hypertension**						
Duration reported% (n)	42.9 (60)	70.9 (56)	53.0 (116)	41.8 (23)	72.7 (24)	53.4 (47)
Mean duration in years (95% CI)	5.3 (4.2–6.3)	6.2 (4.5–7.9)	5.7 (4.8–6.7)	6.0 (3.4–8.6)	6.7 (3.3–10.1)	6.3 (4.3–8.4)
**BP classification%(n)**						
Controlled BP (<140/90 mmHg)	3.6 (5)	25.3 (20)	11.4 (25)	5.5 (3)	33.3 (11)	15.9 (14)
Grade 1 (mild)	45.7 (64)	36.7 (29)	42.3 (93)	45.5 (25)	39.4 (13)	43.2 (38)
Grade 2 (moderate)	32.1 (45)	19.0 (15)	27.4 (60)	21.8 (12)	18.2 (6)	20.5 (18)
Grade 3 (severe)	18.6 (26)	19.0 (15)	18.7 (41)	27.3 (15)	9.1 (3)	20.5 (18)
**BP classification among treated%(n)**						
Controlled BP (<140/90 mmHg)	15.2 (5)	52.8 (19)	34.8 (24)	18.2 (2)	50.0 (8)	37.0 (10)
Grade 1 (mild)	42.4 (14)	27.8 (10)	34.8 (24)	45.5 (5)	25.0 (4)	33.3 (9)
Grade 2 (moderate)	30.3 (10)	8.3 (3)	18.8 (13)	9.1 (1)	18.8 (3)	14.8 (4)
Grade 3 (severe)	12.1 (4)	11.1 (4)	11.6 (8)	27.3 (3)	6.3 (1)	14.8 (4)

Hypertension had been previously detected in 53.4% of hypertensive participants prior to our study, and 31.5% were on antihypertensive treatment at the time of the study. Blood pressure was controlled (<140/90 mmHg) in 11.4% of the hypertensive participants. Women were more likely to have been previously diagnosed, be on treatment and have BP<140/90 mmHg. A greater proportion of women on antihypertensive treatment had BP<140/90 mmHg (52.8%) compared to men on treatment (15.2%). The antihypertensive drugs reported to be taken either alone or in combination by participants included calcium channel blockers (82.6%), β-blockers (18.8%), thiazide diuretics (13.0%) and ACE inhibitors (2.9%).

Table 2 shows the prevalence of the indicators of target organ damage in the study population. Left ventricular hypertrophy (LVH) diagnosed by ECG was observed in 33.3% of the participants. There were patterns of ventricular overload (strain) among 22 out of the 70 with LVH (31.4%). The mean systolic blood pressure was significantly higher (p<0.001) in those with LVH; 168.2 mmHg (SD 24.2 mmHg) compared to those with no LVH; 150.6 mmHg (SD 17.9 mmHg). The mean diastolic blood pressure was also significantly higher (p<0.001) in those with LVH; 101.1 mmHg (SD 14.5 mmHg) compared to those with no LVH; 91.9 mmHg (SD 11.2 mmHg). There was a history of stroke in 2.8% of the participants and 16% had ischaemic changes on ECG.

**Table 2:** Prevalence of hypertensive target organ damage among participants.

CVD risk factor or complication	Definition	Number*	Prevalence (%)
Retinopathy	No retinopathy	62/209	29.8
	Grade 1 retinopathy	118/209	56.5
	Grade 2 retinopathy	27/209	12.9
	Grade 3 retinopathy	2/209	1.0
Left ventricular hypertrophy on ECG	Positive	70/210	33.3
History of stroke	Positive	6/217	2.8
Proteinuria in the absence of nitrites and leucocytes	Negative	169/217	77.8
	Trace	19/217	8.8
	Positive (>300 mg/day)	29/217	13.4
Glomerular filtration rate	<60 ml.min^−1^per 1.73 m^2^	9/219	4.1

* The number of participants was less than the total of 219 where some participants did not complete all investigations.

CKD was present in 4.1% of participants and 13.4% of the participants had albuminuria (>300 mg/d). Hypertensive retinopathy was observed in 70% of participants with 1.0% classified as having Grade 3 retinopathy. None of the participants investigated had hypertensive retinopathy grade IV.

Overall, 104/219 (47.5%) of the participants had evidence of target organ damage. There was no significant age difference (p = 0.13) between those with (51.4 years; SD 7.1 years) and those without (50.0 years; SD 6.0 years) target organ damage. Mean systolic blood pressure was significantly higher (p<0.001) in those with target organ damage (164.7 mmHg; SD 25.2 mmHg) compared to those with no target organ damage (151.1 mmHg; SD 17.3 mmHg). Mean diastolic blood pressure was also significantly higher (p<0.001) among those with target organ damage (100.1 mmHg; SD 14.8 mmHg) compared to those with none (91.8 mmHg; SD 11.0 mmHg).

Table 3 presents the results of multivariate logistic regression analyses modelling the associations between hypertensive target organ damage and selected variables of interest adjusted for potentially confounding variables. There was no significant interaction by sex in the relationships between target organ damage and level of education (p = 0.60), previous detection of hypertension (p = 0.29) and treatment of hypertension (p = 0.60) and the pooled data was presented. The odds of developing target organ damage was lower in women compared to men in all four models (Table 3). The difference was not explained by previous detection and treatment for BP nor the presence of other risk factors such as smoking and diabetes. Adjustment for the current BP level reduced the odds ratio in women slightly (model 3). The participants with the lowest level of education had the highest prevalence of target organ damage, but there was no significant trend observed between the level of education and the development of target organ damage. Previous detection of hypertension did not reduce the odds of developing target organ damage but treatment of hypertension was associated with a lower odds of target organ damage when controlled for previous detection of hypertension. This was however not statistically significant. The participants with Grade 3 hypertension (BP≥180/110 mmHg), had an increased odds of developing target organ damage compared to those with BP<140/90 mmHg (OR; 5.69 CI: 1.46–22.07). The trend increasing BP level - increasing odds of target organ damage, was statistically significant (p = 0.001). The participants diagnosed with diabetes had increased odds of developing target organ damage compared to those with no diagnosis of diabetes. This was however not statistically significant.

**Table 3:** Association between hypertensive target organ damage and selected variables.

	Prevalence of any TOD% (n/N)	Model 1 OR (95% CI)	Model 2 OR (95% CI)	Model 3 OR (95% CI)	Model 4 OR (95% CI)
**Sex**					
Male	59.3 (70/118)	1.00	1.00	1.00	1.00
Female	36.0 (27/75)	0.39 (0.21–0.71)	0.38 (0.20–0.71)	0.32 (0.16–0.65)	0.31 (0.15–0.66)
**Age**					
25–34	50.0 (3/6)	1.00	1.00	1.00	1.00
35–44	58.6 (17/29)	1.28 (0.21–7.86)	1.20 (0.19–7.46)	1.29 (0.20–8.26)	1.18 (0.18–7.69)
45–54	41.8 (41/98)	0.65 (0.12–3.53)	0.61 (0.11–3.37)	0.65 (0.11–3.70)	0.56 (0.10–3.31)
55+	60.0 (36/60)	1.32 (0.23–7.46)	1.22 (0.21–6.99)	1.39 (0.23–8.36)	1.16 (0.18–7.42)
**Detection of BP**					
Newly detected	52.3 (46/88)		1.00	1.00	1.00
Previously detected	48.6 (51/105)		1.31 (0.61–2.82)	0.99 (0.42–2.29)	0.99 (0.42–2.34)
**Treatment of BP**					
No treatment	53.0 (70/132)		1.00	1.00	1.00
Current treatment	44.3 (27/61)		0.77 (0.35–1.73)	1.04 (0.41–2.29)	1.07 (0.42–2.74)
**Hypertension classification (mmHg)**					
BP<140/90	34.8 (8/23)			1.00	1.00
SBP 140–159/DBP 90–99	42.5 (34/80)			0.77 (0.24–2.52)	0.84 (0.25–2.84)
SBP 160–179/DBP 100–109	47.2 (25/53)			0.88 (0.25–3.05)	0.96 (0.27–3.50)
SBP>180/DBP>110	81.1 (30/37)			5.69 (1.46–22.07)	6.14 (1.53–24.6)
p-trend				0.001	0.001
**Level of education**					
Primary	56.5 (35/62)				1.00
Secondary	47.3 (26/55)				1.05 (0.45–2.46)
Tertiary	47.4 (36/76)				0.83 (0.38–1.82)
p-trend					0.62
**Diagnosis of diabetes**					
No diabetes	49.7 (88/177)				1.00
diabetes	56.3 (9/16)				1.45 (0.44–4.78)
**History of smoking**					
No smoking	49.2 (87/177)				1.00
Ex-smoker	57.1 (4/7)				1.08 (0.20–5.94)
Current smoker	66.7 (6/9)				1.11 (0.24–5.19)

Only participants with no missing data were included in the models (N = 193).

Model 1 includes age and sex.

Model 2 includes age, sex, whether previously diagnosed with hypertension and whether on BP lowering treatment.

Model 3: includes age, sex, whether previously diagnosed with hypertension and whether on BP lowering treatment and BP level.

Model 4: includes age, sex, whether previously diagnosed with hypertension, whether on BP lowering treatment, BP level, education, smoking and diabetes.

## Discussion

In this study, we determined the prevalence of hypertensive target organ damage among a group of urban hypertensive civil servants in Ghana, and the factors associated with this. The prevalence of any hypertensive target organ involvement among these civil servants was high (47.5%). The mean systolic and diastolic blood pressures were higher among those with any target organ involvement compared to those without any damage. A significant proportion of participants with left ventricular hypertrophy had strain pattern of ST segment depression and T-wave inversion on ECG which has been reported to be associated with a worse prognosis.[30], [31], [32], [33] Chronic kidney disease was identified in 4% of hypertensive participants and Grade III retinopathy in 1%. None of the participants had Grade IV retinopathy. The odds of developing hypertensive target organ damage was lower in women compared to men and this was partly explained by the current BP level. The odds of developing hypertensive target organ damage was highest in participants with severe hypertension (BP≥180/110 mmHg) and there was a significant positive trend between the Grade of hypertension and the development of target organ damage.

The prevalence of target organ damage, particularly left ventricular hypertrophy and retinopathy, in our study of urban civil servants was comparable to the prevalence reported in people of black African origin from other studies.[34], [35] The specificity of most ECG indexes is reasonably high, but their sensitivity is generally low. Thus even the high levels of left-ventricular hypertrophy observed in our study may have been under-estimated. Black people have been suggested to have more severe forms of arterial hypertension and a greater risk of target organ damage.[36], [37], [38], [39] Stroke is reported to be a major public health problem in SSA where it is reported to be associated with a higher case fatality and to occur at younger ages compared to developed countries.[2], [40], [41], [42] The rather low prevalence of stroke in our study (2.8%) is likely to be largely explained by a “healthy worker effect” since people in employment are less likely to have had strokes. Grades III and IV hypertensive retinopathy (Wegener's classification) were used as evidence of target organ damage in the eyes because of the reported limitations associated with the use of Grades I and II retinopathy.[43] The low prevalence of Grades 3 and 4 hypertensive retinopathy despite poor BP control in this study is similar to findings from other studies conducted in Africa, which have reported a relative rarity of retinopathy on routine fundoscopy even in moderate to severe hypertensive patients.[44]

The odds of developing hypertensive target organ damage were lower in women than men. Women in our study were more likely to have been previously diagnosed, be on treatment and have BP<140/90 mmHg. When we considered only participants on antihypertensive treatment, a greater proportion of women compared to men had their BP controlled (<140/90 mmHg). However the lower odds of target organ damage in women compared to men was not explained by previous detection or treatment of hypertension and was explained only weakly by the BP level. One possible factor that may have contributed to the observed gender difference is the lack of different thresholds for men and women for some of the complications considered (notably ECG changes). Having Grade 3 hypertension was associated with a significantly increased odds of developing target organ damage and there was a significant trend observed between increasing severity of hypertension (determined by BP level) and the odds of developing target organ damage. These findings emphasize the importance of instituting intervention programs on hypertension among civil servants which do not only focus on the primary prevention, early detection and treatment of hypertension, but also on the importance of lowering BP among those with hypertension. Importantly, severe hypertension (BP≥180/110 mmHg) should be prevented. Adequate therapy needed to achieve the goal blood pressure must be initiated and continued. The need to continue treatment despite the absence of symptoms, and the significance of protecting target organs must be emphasized. Of particular concern is the high social and economic burden that would develop from strokes and other cardiovascular complications in the absence of effective programs for blood pressure control.

There is a need to examine the reasons for the poor control of hypertension in this population and particularly in men. Barriers to effective treatment and control of hypertension could include unaffordable drugs, side effects of drugs and ignorance of the consequences of poor control of hypertension. These could possibly explain the high prevalence of hypertensive target organ damage observed in participants with the lowest level of education in this study. Calcium channel blockers were the most common prescribed drugs for hypertension among the participants despite the fact that thiazide diuretics are less expensive. This is possibly due to the concerns that thiazides may be associated with adverse metabolic effects and possibly erectile dysfunction in men.

Most studies reporting the prevalence of target organ damage have been conducted in populations of hypertensive patients seeking medical care with the possibility of capturing a higher frequency of more severe forms of disease than would usually be observed in the general population. This study in a working population may have underestimated the extent of target organ disease in the general population because people with debilitating effects from target organ damage would be under represented in the working group. The high prevalence of target organ involvement observed is thus of great concern and emphasizes a need for development of hypertension control programs aimed at improving the detection of hypertension among civil servants and the entire Ghanaian population, and addressing the issues inhibiting the effective treatment and control of people with hypertension. This is likely to improve the prognosis for people with hypertension.

Our study of hypertensive target organ disease in this population had some limitations. Firstly, our population of civil servants in Accra is unlikely to be representative of the general Ghanaian population, the majority of whom remain in more rural settings and are self employed in farming, trading and other activities. The population of civil servants is however representative of an important section of the urban Ghanaian population from any city in Ghana and indeed in SSA, in any stable formal employment involving non-industrial and predominantly non-manual activities. The sample size was also rather small and may have limited the power to detect significant differences in this population. The findings are however relevant and do provide the evidence needed to conduct a larger scale study among this population. Our investigations for target organ damage did not include echocardiography which is reported to be more sensitive than electrocardiogram in diagnosing left ventricular hypertrophy and predicting cardiovascular risk, neither did we search for microalbuminuria, which is recommended based on evidence to be a sensitive marker of renal damage in hypertension.[25] Electrocardiogram algorithms defining left ventricular hypertrophy have been reported to produce a high false-positive rate in African-Americans and could possibly have overestimated the prevalence in this population of Ghanaian civil servants.[25], [45] The Minnesota criteria have not been validated among Ghanaians and thus the prevalence of left ventricular hypertrophy may have been over (or possibly under) estimated. Black ethnicity has been reported to be associated with an excess of left ventricular hypertrophy using the Minnesota code criteria but we did not compare other commonly used ECG voltage criteria for the identification of left ventricular hypertrophy in our study.[46] We did not control for the duration of hypertension, a potential confounder, in the models because the low level of detection of hypertension in this population and consequently the low level of participants providing information on the duration of hypertension (53%) would have reduced the sample size greatly.

Overall we have demonstrated very high levels of target organ damage among workers with hypertension in urban Ghana associated with poor control of hypertension. Our findings strongly suggest a substantial future burden of both morbidity and mortality from uncontrolled hypertension in Africa. Affordable measures to improve the detection, treatment and control of high blood pressure, and public health measures aimed at reducing blood pressure and cardiovascular risk in the population as a whole are urgently needed.
